# High-fat and High-sucrose Diet-induced Hypothalamic Inflammation Shows Sex Specific Features in Mice

**DOI:** 10.1007/s11064-024-04243-4

**Published:** 2024-09-20

**Authors:** Gabriela C. De Paula, Rui F. Simões, Alba M. Garcia-Serrano, João M. N. Duarte

**Affiliations:** 1 Diabetes and Brain Function Unit, Department of Experimental Medical Science, Faculty of Medicine, Lund University, Lund, Sweden; 2 Wallenberg Centre for Molecular Medicine, Faculty of Medicine, Lund University, Lund, Sweden; 3 Institute for Research in Biomedicine, Bellinzona, Switzerland

**Keywords:** Neuroinflammation, High fat, Sucrose, Reverse diet, Cytokines, Gliosis

## Abstract

Hypothalamic inflammation underlies diet-induced obesity and diabetes in rodent models. While diet normalization largely allows for recovery from metabolic impairment, it remains unknown whether long-term hypothalamic inflammation induced by obesogenic diets is a reversible process. In this study, we aimed at determining sex specificity of hypothalamic neuroinflammation and gliosis in mice fed a fat- and sugar-rich diet, and their reversibility upon diet normalization. Mice were fed a 60%-fat diet complemented by a 20% sucrose drink (HFHSD) for 3 days or 24 weeks, followed by a third group that had their diet normalized for the last 8 weeks of the study (reverse diet group, RevD). We determined the expression of pro- and anti-inflammatory cytokines, and of the inflammatory cell markers IBA1, CD68, GFAP and EMR1 in the hypothalamus, and analyzed morphology of microglia (IBA-1^+^ cells) and astrocytes (GFAP^+^ cells) in the arcuate nucleus. After 3 days of HFHSD feeding, male mice showed over-expression of IL-13, IL-18, IFN-γ, CD68 and EMR1 and reduced expression of IL-10, while females showed increased IL-6 and IBA1 and reduced IL-13, compared to controls. After 24 weeks of HFHSD exposure, male mice showed a general depression in the expression of cytokines, with prominent reduction of TNF-α, IL-6 and IL-13, but increased TGF-β, while female mice showed over-expression of IFN-γ and IL-18. Furthermore, both female and male mice showed some degree of gliosis after HFHSD feeding for 24 weeks. In mice of both sexes, diet normalization after prolonged HFHSD feeding resulted in partial neuroinflammation recovery in the hypothalamus, but gliosis was only recovered in females. In sum, HFHSD-fed mice display sex-specific inflammatory processes in the hypothalamus that are not fully reversible after diet normalization.

## Introduction

Obesity is nowadays considered a 21st century pandemic and has emerged one of the major health risk factors with economic burden [1, 2]. This condition can be defined as excessive adipose tissue accumulation due to an imbalance between energy intake and expenditure [3, 4]. While in genetic obesity a mutation in a gene deregulates energy homeostasis, environmental obesity can be caused by the ingestion of obesogenic diets that are highly caloric, and rich in lipids and sugar [5–7]. The prevalence of obesity is known to be sex-specific, being women more affected than men [1, 2, 8]. This can be due, in part, to the inherent biological difference between the two sexes, in which females have higher body fat proportion compared to males [2]. Sex hormones and menopause also play key roles on obesity development. It has been described that low levels of testosterone can be associated with obesity since this hormone was shown to promote fat consumption [2, 9, 10].

Obesity has been known to induce systemic inflammation that predisposes individuals to the development of comorbidities, including cardiovascular diseases, metabolic syndrome and type 2 diabetes, conditions which in turn impact the brain [11–13]. We have previously shown that long-term exposure to an obesogenic diet rich in saturated fat and sucrose induced reversible alterations in cortex and hippocampus function (behavior) and metabolism in mice [14]. Increased activation of microglia, the brain resident immune cells, was found in the same brain regions, without over-expression of pro-inflammatory cytokines [14], which suggests a sustained low-grade neuroinflammation. In this setting, any cortical and hippocampal alterations were normalized after diet reversal to a low-fat and low-sugar diet [14].

There is increasing evidence that the innate immune activation in the hypothalamus is key element in the pathogenesis of diet-induced obesity. The hypothalamus is the central regulator of body weight and energy homeostasis, integrating and controlling nutrient-sensing signals [15–17]. Hypothalamic inflammation, induced by obesogenic diets, leads to the alteration of normal hypothalamic function that impact feeding behavior and the balance between energy intake and expenditure [18, 19]. Additionally, diet-induced hypothalamic inflammation is typically characterized by the activation of reactive astrocytic and microglial cells, with pro-inflammatory cytokines burden and the subsequent inflammatory cascade [18, 20]. Previous studies using murine models fed high-fat diets (HFD) or high-fat and high-sucrose diets (HFHSD) for different timepoints evidenced this link between inflammation and brain disfunction. A study using blood oxygen level-dependent (BOLD) functional magnetic resonance imaging (fMRI) on mice fed a HFHSD for only 7 days clearly showed hypothalamic dysfunction, namely impaired response to glucose administration [21]. Moreover, HFD-fed mice exhibited an increase in typical astrogliosis and microgliosis markers after 2, 4 and 6 months, including increased density of GFAP and IBA1, and number of GFAP^+^ and IBA1^+^ cells and/or their area [22, 23].

The above-mentioned studies focused on male rodents [18, 20–23]. Male mice fed obesogenic diets are the model of election for metabolic syndrome development. That is because sex-differences on the response to dietary fat are well documented, especially the limited development of insulin sensitivity and hyperinsulinemia in female mice [14, 24]. Thus, little is known on how obesity and metabolic syndrome development during HFD or HFHSD exposure impacts the brain of female rodents, in particular in the hypothalamus. Hypothalamic inflammation differences have been recently reported by Church et al. [25] and Daly et al. [26]. An earlier study has also proposed differential inflammatory profiles in the hippocampus of male and female mice born from HFD-fed dams [27]. These studies further suggest sex differences in the interaction between gut and brain inflammation. In our previous work, the cortex and hippocampus of mice of either sex showed recovery of HFHSD-induced alterations upon diet normalization [14], but it is hitherto unknown whether reversibility of neuroinflammation occurs in the hypothalamus. Using a cohort of mice from this previously reported study [14], we now determined if hypothalamic inflammation induced by HFHSD is a sex-specific reversible process.

## Materials and methods

### Animals

**Fig. 1:**
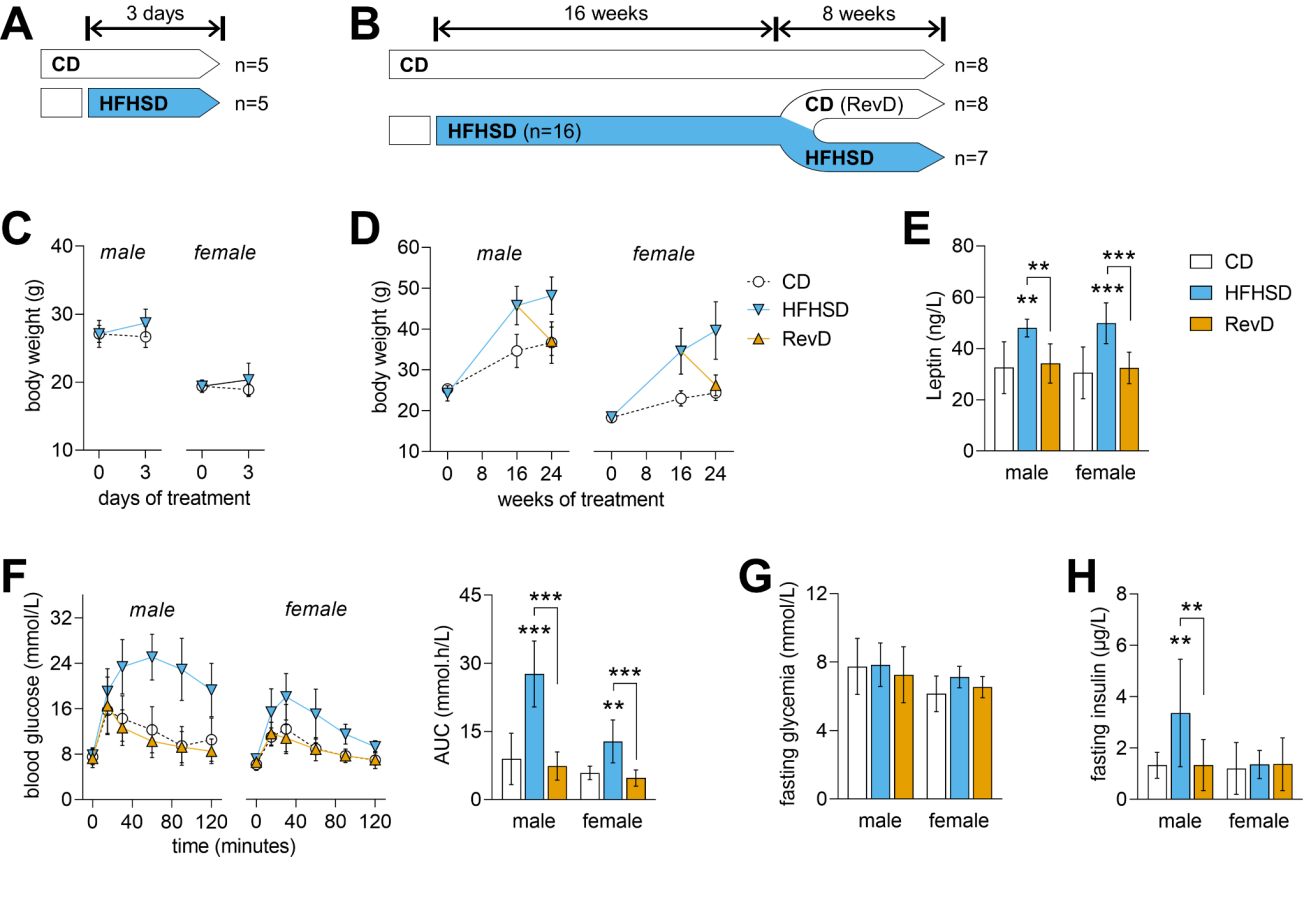
Experimental study design and number of mice (n) per sex in the experimental group (**A**-**B**), body weight (**C**-**D**), and metabolic assessments after 24 weeks of dietary intervention (**E**-**H**). Body weight of male and female mice shows obesity development upon HFHSD feeding, and recovery after diet normalization (RevD) (**D**). Plasma leptin levels after 24 weeks of dietary intervention is increased by HFHSD and normalised in RevD (**E**). Glucose clearance in GTT was reduced by HFHSD feeding, and the area under the curve (AUC) of the GTT shows full recovery after diet normalization (**F**). Fasting glycemia (**G**) and plasma insulin levels (**H**) are indicative of HFHSD-induced insulin resistance in males but not females. Data shown as mean ± SD of *n* = 5-as depicted in (A-B); **P < 0.01, ***P < 0.001 for comparing HFHSD *versus* CD or as indicated

Experiments were performed according to EU Directive 2010/63/EU, approved by the Malmö/Lund Committee for Animal Experiment Ethics (#994/2018), and are reported following the ARRIVE guidelines (Animal Research: Reporting in Vivo Experiments, NC3Rs initiative, UK). 8-weeks old male and female C57BL/6J mice (RRID: IMSR_JAX:000664) were purchased from Taconic Biosciences (Köln, Germany), and housed in groups of 3–5 animals on a 12 h light-dark cycle with lights on at 07:00, room temperature of 21–23 °C and humidity at 55–60%. Mice were habituated to the facility for 1 week upon arrival and subsequently randomly assigned to 5 experimental groups receiving: (i) a 10%-fat control diet (CD) for 3 days, (ii) a CD for 24 weeks, (iii) a composition-matched high-fat diet (60%-fat) plus access to a 20%(w/v) sucrose in drinking water (HFHSD), for 3 days, (iv) a HFHSD for 24 weeks, or (v) a reversed diet (RevD), that consisted of HFHSD feeding for 16 weeks followed by CD feeding for 8 weeks (Fig. 1A-B). Mice receiving HFHSD also had access to sugar-free water. Food and water were provided *ad libitum*.

Diets were acquired from Research Diets (New Brunswick, NJ-USA): a high-fat lard-based diet with 60% kcal of fat (D12492) and a control diet containing 10% kcal of fat (D12450J), with total energy of 5.21 and 3.82 kcal/g, respectively [14].

After 24 weeks, we carried out glucose tolerance tests (GTT) and plasma hormone analyses as detailed previously [14]. Brain samples were collected at 3 days or 24 weeks of diet intervention after decapitation under brief isoflurane anesthesia.

### Glucose Tolerance Test

Mice were fasted for 6 h and basal blood glucose was measured from tail tip blood with the Accu-Chek Aviva glucometer (Roche, Manheim, Germany). Then, mice were given 2 g/kg glucose intraperitoneally (i.p.) from a 30% (w/v) solution in 0.9% saline, and blood glucose was measured from the tail tip at 15, 30, 60, 90 and 120 min after injection.

### Insulin and Leptin Quantification

Commercially available mouse ELISA kit were used to quantify plasma insulin (#10-1247-10, Mercodia, Uppsala, Sweden; RRID: AB_2889906) and leptin (#ab100718, Abcam, Cambridge, United Kingdom; RRID: AB_2889903) levels, and were measured in accordance with the manufacturer’s instructions.

### Real-time Polymerase Chain Reaction (RT-PCR) for mRNA Expression

RNA was isolated from mice hypothalamus with Trizol (#15596026, Invitrogen, USA), and reverse transcribed using the script cDNA SuperMix (#95048, Quantabio, England), and then the resulting cDNA was used for quantitative RT-PCR as detailed by [28] using PerfeCTa SYBRgreen SuperMix (#95054, Quantabio, England) and primers for allograft inflammatory factor 1 (IBA1), cluster of differentiation 68 (CD68), adhesion G protein-coupled receptor E1 (EMR1), glial fibrillary acidic protein (GFAP), interleukin (IL)-1β, IL-6, IL-10, IL-13, IL-18, interferon γ (IFN-γ), transforming growth factor β (TGF-β), tumor necrosis factor α (TNF-α), and β-actin (Table 1). Gene expression was normalized to β-actin expression with the comparative cycle threshold method (ΔΔCT).

**Table 1:** Primers used for real time-PCR, respective gene, accession number, annealing temperature (T_a_), and measured primer size

Target gene	Accession number	Forward primer (5’→3’)	Reverse primer (3’→5’)	T_a_ (°C)	Product size (base pairs)
*CD68*	NM_001291058.1	GGCGGTGGAATACAATGTGTCC	AGCAGGTCAAGGTGAACAGCTG	60	156
*EMR1*	NM_001355722.1	CGTGTTGTTGGTGGCACTGTGA	CCACATCAGTGTTCCAGGAGAC	60	133
*GFAP*	NM_001131020.1	AACGACTATCGCCGCCAACTG	CTCTTCCTGTTCGCGCATTTG	60	99
*IBA1*	NM_001361501.1	TGGAGTTTGATCTGAATGGCAATG	AGCCACTGGACACCTCTCTA	60	126
*IFN-γ*	NM_138880.3	CAGCAACAGCAAGGCGAAAAAGG	TTTCCGCTTCCTGAGGCTGGAT	60	145
*IL-10*	NM_012854.2	CGGGAAGACAATAACTGCACCC	CGGTTAGCAGTATGTTGTCCAGC	60	130
*IL-13*	NM_053828.1	AACGGCAGCATGGTATGGAGTG	TGGGTCCTGTAGATGGCATTGC	60	104
*IL-18*	NM_019165.2	GACAGCCTGTGTTCGAGGATATG	TGTTCTTACAGGAGAGGGTAGAC	60	159
*IL-1β*	NM_031512.2	TGGACCTTCCAGGATGAGGACA	GTTCATCTCGGAGCCTGTAGTG	60	145
*IL-6*	NM_012589.2	TACCACTTCACAAGTCGGAGGC	CTGCAAGTGCATCATCGTTGTTC	60	116
*L14*	NM_025974.2	GGCTTTAGTGGATGGACCCT	ATTGATATCCGCCTTCTCCC	60	145
*TGF-β*	NM_021578.2	AAGAAGTCACCCGCGTGCTA	TGTGTGATGTCTTTGGTTTTGTCA	60	70
*TNF-α*	NM_012675.3	GGTGCCTATGTCTCAGCCTCTT	GCCATAGAACTGATGAGAGGGAG	60	139
*β-Actin*	NM_007393.5	AGCCATGTACGTAGCCATCC	CTCTCAGCTGTGGTGGTGAA	60	228

### Immunofluorescence Confocal Microscopy

Mice were sacrificed under isoflurane anesthesia by cardiac perfusion with cold PBS and then cold phosphate-buffered formaldehyde (Histolab, Askim, Sweden), and brains were cryosectioned into 20 μm slices [29]. Immunolabeling was carried out as detailed previously [21] with the primary antibodies: rabbit anti-allograft inflammatory factor 1 (IBA1, dilution 1:200; #019-19741, Fujifilm Wako, Japan; RRID: AB_839504), and anti-glial fibrillary acidic protein (GFAP) pre-tagged with AF488 (dilution 1:500; #53-9892-82, ThermoFisher Scientific, Göteborg, Sweden; RRID: AB_10598515). Secondary antibody (dilution 1:500) was from ThermoFisher: AF568-conjugated goat anti-Rabbit IgG (#A-21069; RRID: AB_141416). After mounting, slices were examined under a Nikon A1RHD confocal microscope (Nikon Instruments, Tokyo, Japan). Images were acquired with NIS-element v5.20.01 (Laboratory Imaging, Nikon), and analyzed in ImageJ (NIH, Bethesda, MD, USA; RRID: SCR_003070) as previously [14].

### Statistics

Results are presented as mean ± SD unless otherwise stated. Partial least-squares (PLS) regression with 5 components was applied on z-scores of gene expression profiles using MATLAB 2019a (MathWorks, Natick, MA-USA; RRID: SCR_001622). The PLS model was fit to each CD-HFHSD paired dataset, and the variable importance in projection (VIP) was calculated for each gene. Data from the RevD group was not analyzed but reconstructed with the obtained models for male and female mice. One-sample t-tests were used for determining specific gene expression changes. Prism 9.4.0 (GraphPad, San Diego, CA-US; RRID: SCR_002798) was used for analysis of metabolic phenotype and immunofluorescence results. After assessing normality with the Kolmogorov-Smirnov and Shapiro-Wilk tests, data were analyzed with the two-way ANOVA followed by independent comparisons with the Fisher’s least significant difference (LSD) test. One-way ANOVA was also used to compare the effect of diet on males and females separately on immunofluorescence analysis. Statistically significant differences were considered for *P* < 0.05.

## Results

As reported previously, HFHSD feeding induces obesity with glucose intolerance in both genders, and hyperinsulinemia in male but not female mice [14]. In particular, 3 days of HFHSD exposure was sufficient to induce a small weight increase (Fig. 1C; weight gain ANOVA: gender *P* = 0.396, diet *P* < 0.001, interaction *P* = 0.533), which is pronounced after 24 weeks, and recovers upon diet normalization (Fig. 1D; weight gain ANOVA: gender *P* < 0.001, diet *P* < 0.001, interaction *P* < 0.001). In line with obesity and increased fat deposition [14], HFHSD but not RevD mice showed increased fed leptin in plasma (Fig. 1E; ANOVA: gender *P* = 0.820, diet *P* < 0.001, interaction *P* = 0.800). Male mice fed the HFHSD showed more severe glucose intolerance than females in a GTT (Fig. 1F; ANOVA: gender *P* < 0.001, diet *P* < 0.001, interaction *P* < 0.001). HFHSD had negligible effects on fasting glycaemia, although females showed lower blood glucose than males (Fig. 1G; ANOVA: gender *P* = 0.006, diet *P* = 0.330, interaction *P* = 0.510), while fasting insulinaemia was impacted by HFHSD in males but not females (Fig. 1H; gender *P* = 0.029, diet *P* = 0.042, interaction *P* = 0.018). Finally, diet normalization after 16 weeks of HFHSD exposure resulted in recovery of body weight, glucose tolerance, and hyperinsulinemia (in males) to control values (Fig. 1).

### Effect of HFHSD-feeding for 3 days on Hypothalamic Inflammation Markers

**Fig. 2:**
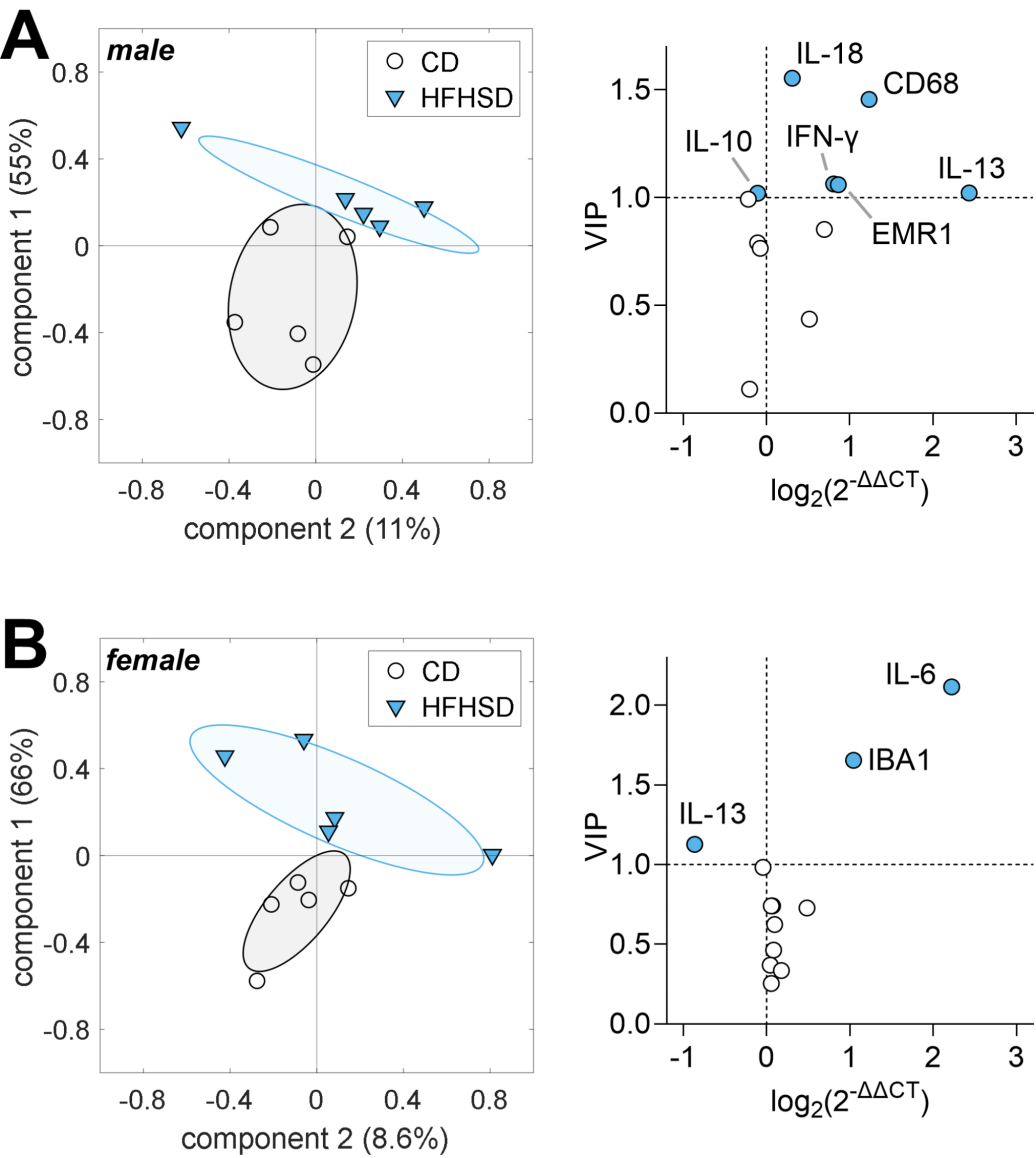
HFHSD feeding for 3 days induced gender-specific hypothalamic inflammation, that is, differential gene expression changes were observed in male (**A**) and female (**B**) mice. Graphs on the left show mouse grouping for components 1 and 2 of the PLS regression. Individual mice are represented by symbols, and group SD by ellipsoids. Variance explained by each component is shown in parenthesis. Graphs on the right show fold-change of gene expression, and VIP scores calculated from the resulting PLS model. Filled symbols represent VIP > 1. Data shown as mean ± SD of *n* = 5 for each group

Thaler et al. reported hypothalamic inflammation within a few days after HFD exposure in mice [18]. Thus, we first analyzed mRNA expression levels of pro-inflammatory cytokines IL-1β, IL-6, IL-18, TNF-α and IFN-γ, anti-inflammatory cytokines IL-10, IL-13 and TGF-β, as well as astrogliosis (GFAP) and microgliosis (IBA1, CD68 and EMR1) markers in the hypothalamus of male and female mice after 3 days HFHSD feeding. A PLS regression with 5 components provided a separation of mice in HFHSD or CD based on their gene expression (Fig. 2) and explained 98% and 95% of the variance in males and females, respectively. Notably, HFHSD feeding for 3 days triggered different inflammatory responses in male and female mice. Namely, male mice showed increased expression of IL-13, IL18, IFN-γ, CD68 and EMR1 and a small decrease of IL-10 (Fig. 2A), while female mice had increased expression of IL-6 and IBA1, and reduced expression of IL-13 (Fig. 2B).

### Cytokine Expression after 24 Weeks of HFHSD-feeding and Diet Normalization

**Fig. 3:**
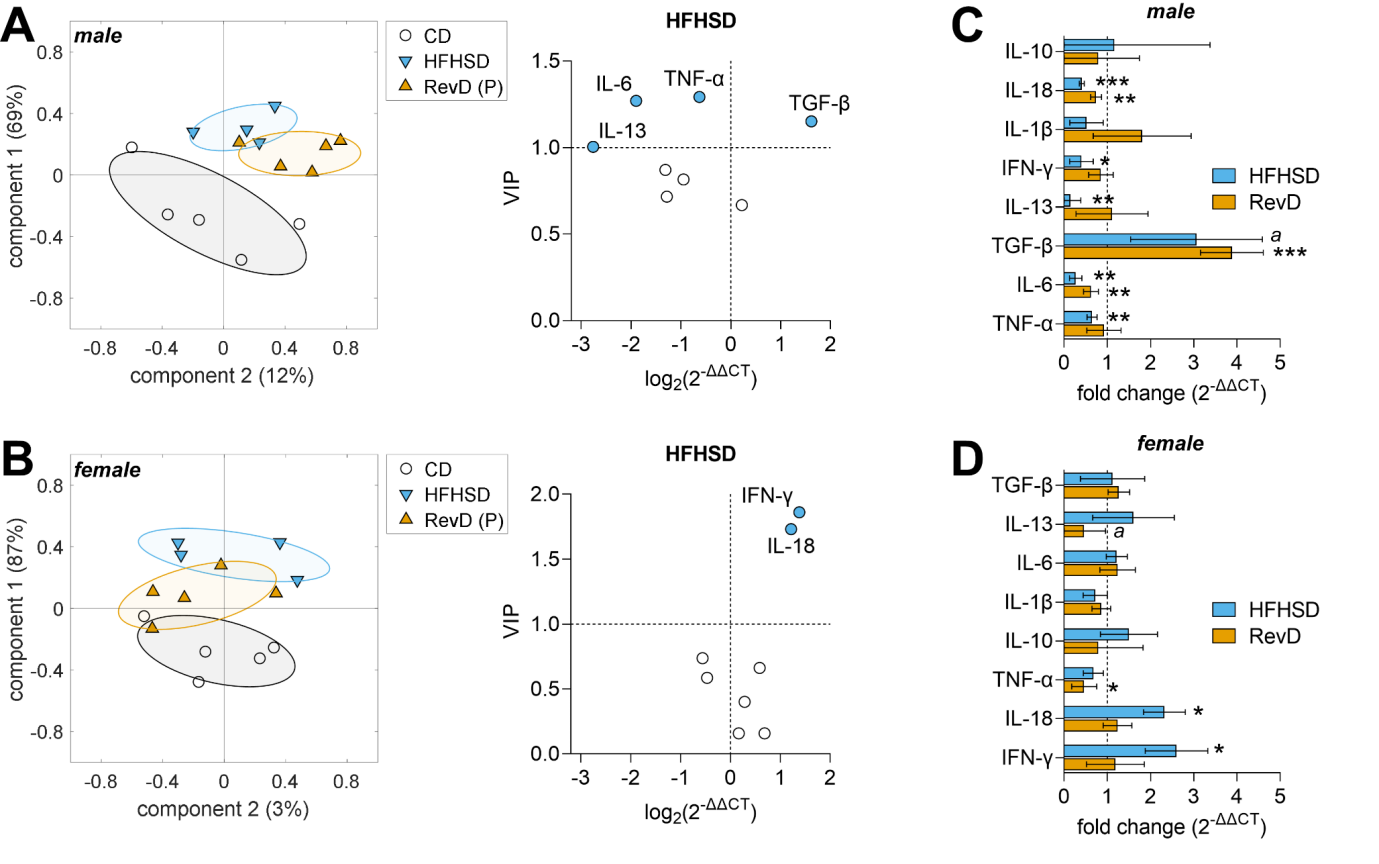
HFHSD feeding for 24 weeks induced gender-specific hypothalamic inflammation, that is, differential gene expression changes were observed in male (**A**) and female (**B**) mice. PLS regression models were constructed for HFHSD and CD, and then used to calculate the component space for predicting effects of diet normalization in RevD (P). Graphs on the left show mouse grouping for components 1 and 2 of the PLS regression. Individual mice are represented by symbols, and group SD by ellipsoids. Variance explained by each component is shown in parenthesis. Graphs on the right show fold-change of gene expression for HFHSD and RevD relative to CD, and VIP scores calculated from the resulting PLS model. Filled symbols represent VIP > 1. Panels C and D show gene expression in HFHSD and RevD mice relative to control CD (mean ± SD of *n* = 5 for each group). Crescent VIP scores are represented from top to bottom. Significance for one sample t-test comparisons to 1 are indicated as follows *P < 0.05, **P < 0.01, ***P < 0.001 (^a^*P* = 0.07)

We next analyzed mice hypothalamic cytokine expression after 24 weeks of HFHSD. A PLS regression with 5 components provided a separation of mice in HFHSD or CD based on their gene expression (Fig. 3A-B) and explained 92% and 98% of the variance in males and females, respectively. Like 3 days of HFHSD, prolonged HFHSD feeding resulted in different cytokine expression profiles in each gender. Male mice presented an overall tendency for a decrease in pro- and anti-inflammatory cytokine mRNA expression after HFHSD, relative to CD, with important decreases in expression of TNF-α, IL-6 and IL-13, and an increase in TGF-β (Fig. 3A). In contrast, female mice on HFHSD showed generally unchanged cytokine expression, with increases in the expression of pro-inflammatory cytokines IL-18 and IFN-γ (Fig. 3B). The PLS regression models were then used to predict effects of diet normalization in mice of the RevD group. While the PLS component space indicates a cytokine expression shift in RevD mice, there was no full normalization of the cytokine profile in male (Fig. 3A) or female (Fig. 3B) mice. Only a few cytokines normalized their expression after diet reversal in male mice, such as TNF-α and IL-13 (Fig. 3C). Interestingly, while HFHSD-induced overexpression of IL-18 and IFN-γ were normalised in females, other cytokines appeared modified after diet reversal, namely TNFα (Fig. 3D).

### Gliosis in the Hypothalamic Arcuate Nucleus

**Fig. 4:**
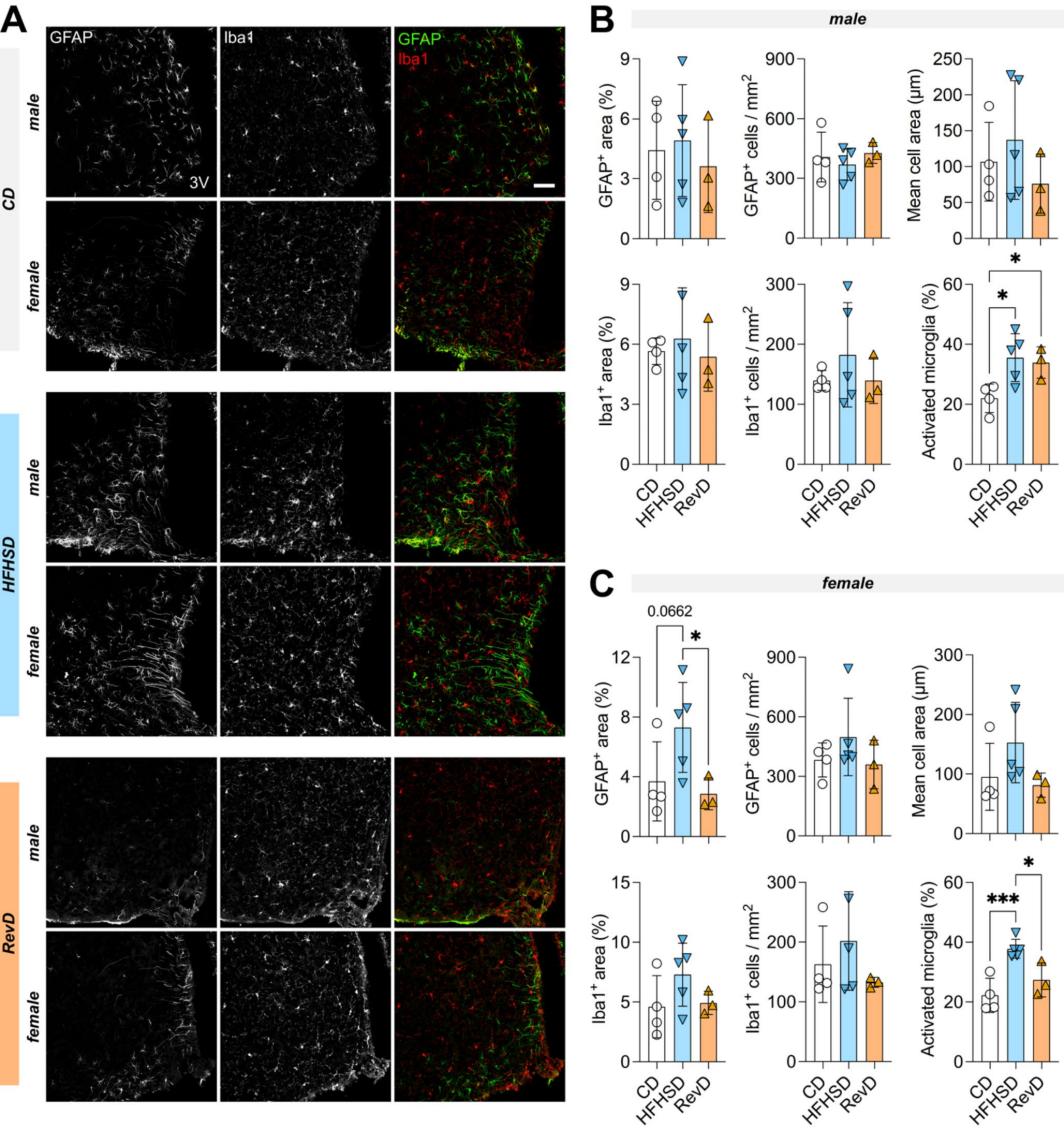
Astrogliosis and microgliosis in the arcuate nucleus of male and female mice. (**A**) representative micrographs of astrocytes (GFAP^+^ cells, green) and microglia (IBA1^+^ cells, red) cells in the arcuate nucleus (scale bar is 50 μm). Total GFAP stained area, number of GFAP^+^ cells or mean cell area were evaluated for astrogliosis while IBA1 stained area, number of IBA1^+^ cells or fraction of activated microglia (poorly ramified cells) were evaluated in male (**B**) and female (**C**) mice. Bars are mean ± SD of *n* = 4 (CD), *n* = 5 (HFHSD), *n* = 3 (RevD), and symbols represent individual mice. *p < 0.05, ***P < 0.001 from Fisher’s LSD test after significant ANOVA

We next analyzed the morphology of astrocytes (GFAP^+^ cells) and microglia (IBA-1^+^ cells) in the arcuate nucleus (ARC) of hypothalamus by immunofluorescence imaging in mice fed with HFHSD for 24 weeks (Fig. 4A). We analyzed astrocytes by the number, total, and mean area of GFAP^+^ cells; and microglia by the total number and area of IBA1^+^ cells, as well as the percentage of activated microglia as morphologically defined elsewhere [30]. No major alterations were found between groups regarding GFAP immunostaining on cell number and area in male mice (Fig. 4B; ANOVA: diet *P* = 0.6747 number; *P* = 0.7956 area). In female mice, on the other hand, there was a tendency for increased GFAP area (ANOVA: diet CD versus HFHSD *P* = 0.0662), which was normalized by diet reversal (Fig. 4C; ANOVA: diet HFHSD versus RevD *P* < 0.05). While no major alterations were found between groups regarding total Iba1^+^ cell number and area, an increase on activated microglia with HFHSD was found in both male (ANOVA: diet *P* < 0.05) and female mice (Fig. 4B-C; ANOVA: diet *P* < 0.001). While microglia remain activated when male mice were switched to control diet (Fig. 4B; ANOVA: gender *P* = 0.8187, diet *P* = 0.7418, interaction *P* = 0.6901), microglia activation was reversed in females (Fig. 4C; ANOVA: diet *P* < 0.05).

## Discussion

Located in the mediobasal hypothalamus, the ARC is particularly positioned to sense circulating factors that regulate metabolism. Together with the median eminence, the ARC is a circumventricular organ that lacks a true blood–brain barrier (BBB) formed by endothelial cells, resulting in exposure to circulating factors [31]. Others have previously reported effects of obesogenic diets in the development of hypothalamic neuroinflammation [18–20, 22]. However, whether this is a sex-specific, reversible process still needs validation. In this study, we found that female mice fed HFHSD for 24 weeks exhibited a shift in cytokine mRNA levels, accompanied by astrocyte and microglia activation. These features were largely normalized to control levels after diet reversal. On the other hand, male mice presented an overall decrease in pro- and anti-inflammatory cytokine levels, which also tended to be normalized after diet reversal. Interestingly, female but not male mice showed astrogliosis after long-term HFHSD exposure. Since only females recovered microglia activation, we propose that astrogliosis in long-term HFHSD exposure is not triggering injury, but can rather facilitate the resolution of neuroinflammation. Antagonic data regarding sex-specific diet-induced astrogliosis can be found in the literature. In contrast to our findings, a shorter 8-week HFD exposure was found to induce astrogliosis in the arcuate nucleus of males but not females [32].

It has been previously reviewed that both basal body inflammatory and immune responses have sex-specific differences based on genetic mediators (such as sex chromosomes and microRNAs and long non-coding RNAs), hormonal mediators (estradiol, progesterone, and androgens), and environmental mediators (e.g. nutrition and microbiome) [33]. It was formerly found that both innate and adaptive responses are generally higher in females compared to males. Females tend to have greater antibody levels and responses, higher immunoglobulin levels and higher B cell numbers [34, 35]. For example, it has been shown that female adult mice possess higher levels of T helper 1 cytokine producing cells, responsible for the production of INF-γ [36], which corroborates our findings. This gender-specific differences can be due to the fact that both androgen and estrogen response elements can be found in the promoters of several innate immunity genes, leading to a dimorphic immune response [37]. It was already shown that low levels of the female sex hormone estradiol can increase the production of pro-inflammatory cytokines IL-1, IL-6 and TNF-α, while higher levels of this hormone have the opposite effect [38]. On the other hand, male sex hormones androgens have been described to exhibited anti-inflammatory properties. Testosterone was revealed to increase the levels of anti-inflammatory cytokine TGF-β [39], while reducing the levels of pro-inflammatory cytokine TNF-α [40], as observed in the present study.

Few preclinical studies have looked at the effect of obesogenic diets comparing male and female, and even fewer took this gender consideration regarding hypothalamic inflammation. Daly et al. investigated sex differences when mice were fed with HFHSD and found that male mice displayed lower levels of the pro-inflammatory cytokines IL-1β and IL-6 compared sex-related mice fed a low-fat, low-sucrose diet (LFLSD) [26]. Contrarily, HFHSD fed female mice evidenced an increase in cytokines levels compared to the correspondent LFLSD group [40]. The authors found no alterations on TGF-β and TNF-α protein levels. Recently, a broad study comparing sex- and age- dependent behavior and inflammatory parameters in mouse under high-fat but not high sucrose diet, described alterations in plasma of young female mice, while no effects after 5–6 months of HFD were observed on male mice [41]. Increased pro-inflammatory cytokines and chemokines such as IL17A/CTLA8, Eotaxin/CCL11, MCP3/CCL7, and Leptin were observed in HFD-fed females, with decreased levels of IL22 (the IL-10 family cytokine that is produced by T cells) [41]. In the brain innate immunity, sex-specific differences are found to show females more resistant to hypothalamic inflammation [42, 43]. Dorfman et al. describe the hypothalamic microglial activation of mice fed HFD as part of the CX3CL1-CX3CR1 pathway [42]. In females, the increase in CX3CR1 signaling triggered by HFD protects against diet-induced obesity by reducing microglial activation. Increased number and size of the female brain immune cells was also detected in an obese model induced by Olanzepine, with the gliosis associated with higher levels of TNF-α within the hypothalamus [43]. This goes in contrast with the decrease in microglial cell complexity in HFHSD male mice found by Daly et al., with no changes observed between the female mice groups.

Dietary and nutraceutical interventions targeting the gut microbiome and brain health are emerging areas of research aimed at addressing neuroinflammation. Approaches that focus on correcting the dysbiosis caused by poor dietary habits may help to halt or at least reduce the immune and inflammatory responses that contribute to the condition. Interestingly, the same study of Daly et al. described above reported major differences in gut microbiome species between all the different groups, and, more specifically, HFHSD male mice develop an increase gut microbiota species diversity compared to LFLSD. Moreover, a correlation between diet-induced gut microbiome alterations and hypothalamic inflammatory profile was evident [26]. This dysbiosis was previously shown to affect the central nervous system physiology and inflammation through the gut-brain axis, that encompasses a panoply of intricate pathways that include the vagal nerve, the immune system, and bacterial-derived metabolites [44, 45]. Since neuroinflammation can be a direct response to how components of the diet are metabolized after digestion and that many metabolites can specifically arise from gut microbiome metabolism, one can speculate that gut dysbiosis can be a major player in diet-induced neuroinflammation. Intestinal inflammation and increased permeability develop in adult male mice after 12 weeks of HFD or high sugar diet [46], with a clearly variable diet-dependent changes in the levels of cytokines in the colon of mice [25, 46]. However, more studies are needed on the sex-related alterations across the gut-brain axis and its connection between gut dysbiosis as a cause for neuroinflammation. Moreover, little is known about the effects of a RevD on the gut microbiome.

To our surprise, neuroinflammatory markers measured in our study were not strikingly increased after 3 days of HFHSD exposure, in contrast to observations by Thaler et al. using HFD [18]. Aside any possible experimental peculiarities on mouse strain, diet composition, housing or handling, our experience feeding obesogenic diets [14, 22, 23, 47] to mice leads us to believe that HFD alone might be a stronger inducer of metabolic syndrome than HFHSD. The lower severity of metabolic syndrome during HFHSD than during HFD is likely the reason for the present study to not reproduce the early hypothalamic inflammation reported for HFD-fed mice [18, 22, 23].

To conclude, mice fed HFHSD display complex sex-specific changes of inflammatory cytokine profiles in the hypothalamus that can be partially reversed by diet normalization. These cytokine changes are, however, not necessarily accompanied by or indicative of gliosis. In fact, male mice showed activation of microglia but not astrocytes upon HFHSD feeding, while female mice showed activation of both, and gliosis was reversible in females but not males.

## Data Availability

All data are contained within the manuscript and can be shared upon request to the corresponding author.
